# Characterization of Chicken Spleen Transcriptome after Infection with *Salmonella enterica* Serovar Enteritidis

**DOI:** 10.1371/journal.pone.0048101

**Published:** 2012-10-19

**Authors:** Marta Matulova, Jana Rajova, Lenka Vlasatikova, Jiri Volf, Hana Stepanova, Hana Havlickova, Frantisek Sisak, Ivan Rychlik

**Affiliations:** Veterinary Research Institute, Brno, Czech Republic; University of Osnabrueck, Germany

## Abstract

In this study we were interested in identification of new markers of chicken response to *Salmonella* Enteritidis infection. To reach this aim, gene expression in the spleens of naive chickens and those intravenously infected with *S*. Enteritidis with or without previous oral vaccination was determined by 454 pyrosequencing of splenic mRNA/cDNA. Forty genes with increased expression at the level of transcription were identified. The most inducible genes encoded avidin (AVD), extracellular fatty acid binding protein (EXFABP), immune responsive gene 1 (IRG1), chemokine ah221 (AH221), trappin-6-like protein (TRAP6) and serum amyloid A (SAA). Using cDNA from sorted splenic B-lymphocytes, macrophages, CD4, CD8 and γδ T-lymphocytes, we found that the above mentioned genes were preferentially expressed in macrophages. AVD, EXFABP, IRG1, AH221, TRAP6 and SAA were induced also in the cecum of chickens orally infected with *S*. Enteritidis on day 1 of life or day 42 of life. Unusual results were obtained for the immunoglobulin encoding transcripts. Prior to the infection, transcripts coding for the constant parts of IgM, IgY, IgA and Ig light chain were detected in B-lymphocytes. However, after the infection, immunoglobulin encoding transcripts were expressed also by T-lymphocytes and macrophages. Expression of AVD, EXFABP, IRG1, AH221, TRAP6, SAA and all immunoglobulin genes can be therefore used for the characterization of the course of *S*. Enteritidis infection in chickens.

## Introduction

Except for the infection with *S. enterica* serovars Gallinarum or Pullorum, chicken infection with the other remaining *S. enterica* serovars is usually not associated with any obvious clinical signs. Despite the absence of gross clinical signs, chickens respond to oral infection by an inflammatory response associated with heterophil and monocyte/macrophage infiltration into the cecal mucosa. The scope of this response is age dependent and is more obvious in chickens up to 2 weeks of age than in adult birds [1]. In agreement with this, proinflammatory cytokines such as IL1β, IL6, IL17 and IL22, together with IFNγ and iNOS are induced in the cecum after infection, either by epithelial cells, resident phagocytes, or infiltrating phagocytes or lymphocytes [2]–[4]. A similar cytokine gene expression can be recorded also in the spleen, although the induction rates in the spleen after oral infection are usually lower than those observed in the cecum [5]. The low response of splenic leukocytes to *S. enterica* infection can be overcome by intravenous infection. The chicken response to intravenous infection with *S. enterica* is characterized by splenomegaly associated with macrophage and heterophil infiltration and Th1 and Th17 cytokine signaling, similar to the response in the cecum after oral infection [4], [5].

Another puzzling phenomenon is that the immune response of naive or vaccinated chickens to *S. enterica* infection is the same in terms of a qualitative response. So far the only described differences are mainly in quantitative expression of the immune response – the vaccinated chickens respond to *S. enterica* infection by lower cellular infiltrates and lower proinflammatory cytokine signaling than the naive chickens [1], [6]. This conclusion is valid for both the cecum after oral infection and the spleen after intravenous infection [5].

However, there is at least one difference between the oral and intravenous challenge; namely the production of anti-LPS antibodies. Orally infected chickens produce quite low anti-LPS antibodies whilst intravenous challenge leads to an extremely high antibody production which, unlike the oral challenge, is independent of previous contact with the antigen, i.e. the vaccination status [5]. The reason for a high and rapid antibody production is rather unclear since B-lymphocytes and antibody production are considered as dispensable for the chicken's defense against *S. enterica* infection [6].

In the search for markers for the protection of vaccinated chickens against *S*. Enteritidis infection we used the model of intravenous infection. We hypothesized that if the spleen sizes differ between the vaccinated and infected, naive and infected and non-infected chickens [4], [5], there must be significant differences in gene expressions among these 3 groups of chickens. We therefore purified mRNA from the spleens of intravenously infected chickens and subjected it to transcriptome characterization by 454 pyrosequencing. This approach resulted in an unbiased identification of chicken genes which are induced in response to systemic *S. enterica* infection. In addition, we have shown that some of the newly identified genes were induced also in the cecum of orally infected chickens. However, chickens which had been vaccinated prior to the challenge did not induce these genes in the cecum after oral challenge which in turn can be used as a marker of vaccine efficacy and specific immunity to *S*. Enteritidis.

## Results

### Expression in the spleen

Pyrosequencing resulted in 140,827 reads when cDNA from the spleen of the non-infected chicken was sequenced, 100,971 reads from the spleen of the chicken after *S*. Enteritidis infection and 53,762 reads from the spleen of the chicken which had been vaccinated prior to the infection. Average read size was 426 bp. Considering 1,000 bp as an average gene size and 20–23,000 genes forming chicken genome [7], total chicken transcriptome represents approx. 20–23 Mb of mRNA sequence. We therefore achieved approx. 1× coverage for the transcriptome of the spleen of vaccinated chicken, 2× coverage for the infected spleen transcriptome and 3× coverage for the non-infected spleen transcriptome. This means that we identified only the highly expressed genes and many low level expressed genes, despite their differential expression in the spleen of infected or non-infected chickens, might remain undetected.

Combining all 3 samples in the *de novo* assembly resulted in the identification of 8,844 isotigs which were subjected to Blast2GO analysis. After the analysis, the number of expressed genes decreased to 6,633 transcripts because some of the isotigs were identical to different parts of the same genes (Tab. S1). After applying all the quality selective criteria, 23,663 reads from the spleen of the non-infected chicken, 21,442 reads from the spleen of the infected chicken and 18,536 reads from the spleen of the vaccinated and infected chicken were finally included in the quantification of expression (the majority of the excluded transcripts comprised of rRNA, polyA sequences or repeated sequences). For 99 and 78 genes we predicted that these might be down- or upregulated in the spleen after i.v. *S*. Enteritidis infection, respectively (Tab. S2 and Tab. S3). Similar results were observed also when we performed BLASTX analysis of all individual reads against chicken genome only (data not shown).

**Figure 1:**
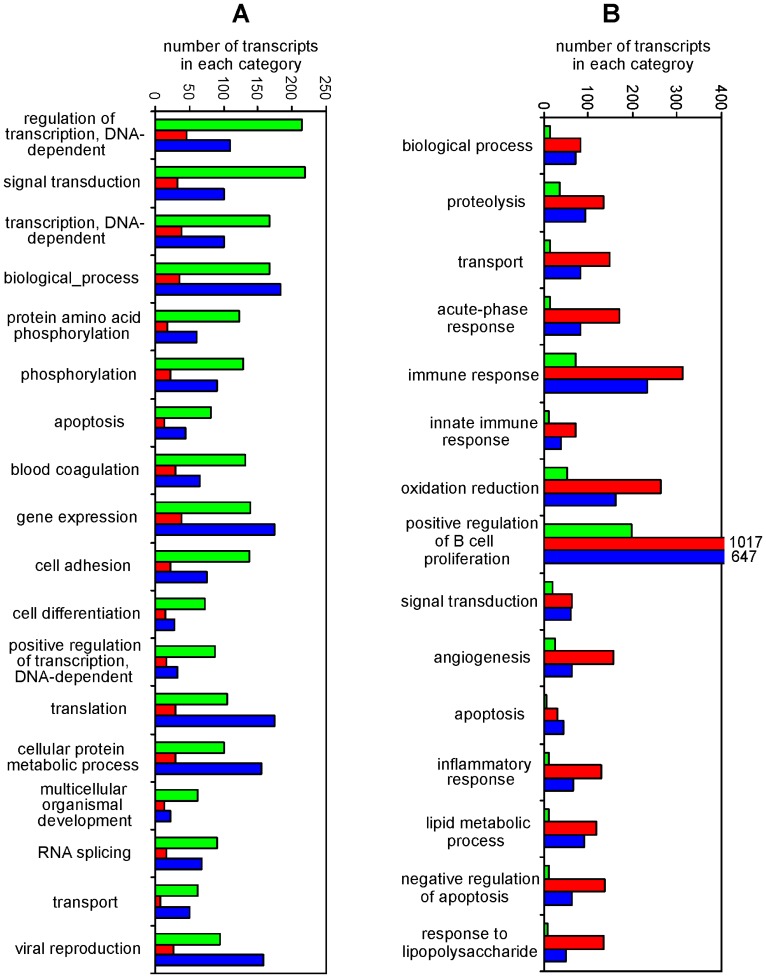
Cellular processes suppressed or induced in the spleen after *S*. Enteritidis infection. Green columns – expression in the spleen of non-infected chickens, red – expression in the spleen of infected chickens, blue – expression in the spleen of vaccinated and infected chickens. Number of transcripts for “positive regulation of B-cell proliferation” exceeding the Y-axis scaling is shown numerically. Panel A, suppressed Cellular processes, Panel B induced cellular processes.

When gene ontology classification on cellular processes was retrieved for these transcripts, the downregulated transcripts were classified as involved in transcription, translation, signal transduction, phosphorylation, cell adhesion and differentiation (Fig. 1). The transcripts upregulated after the infection were classified as associated with proteolysis, cellular transport, acute-phase response, response to LPS, innate immune and inflammatory response, lipid metabolic processes, angiogenesis and apoptosis (Fig. 1). As can be seen in Fig. 1, the suppression of gene expression was always lower in the vaccinated chicken than in the non-vaccinated chicken after the challenge, and processes like gene expression or translation were not affected in the vaccinated chicken at all (the same or even higher number of transcripts in the vaccinated and infected chicken as in the non-infected control). Similarly, except for cellular transport, the cellular processes upregulated after *S*. Enteritidis challenge were always expressed at a higher level in the non-vaccinated chicken when compared with the chicken which was vaccinated prior to the infection (Fig. 1).

In the next step we designed real time PCRs to verify the expression of all 78 genes predicted as upregulated after i.v. infection with *S*. Enteritidis (we did not elaborate on genes suppressed after *S*. Enteritidis in this study any further). Significant upregulation, either in the non-vaccinated or vaccinated chickens, was confirmed in 40 of them (Tab. 1). When we compared the expression of individual genes in the spleens of naive or vaccinated chickens after i.v. challenge, 18 genes were expressed differently although the difference only rarely exceeded a factor of 2 (Tab. 1).

**Table 1:** Fold increase in gene expression in chickens after *S*. Enteritidis infection determined by real-time PCR.

		Spleen		Cecum		
		Day 46		Day 4	Day 46	
gene	transcript	Inf	Vacc	Inf	Inf	Vacc
AH221	chemokine ah221	7.68	6.00	11.38	3.10	1.04
ANG	angiogenin / ribonuclease A	2.70	2.41	3.33	2.76	1.15
ANXA2	annexin A2	4.17	2.77	0.80	0.91	0.97
ASAH	acid ceramidase	2.35	1.67	0.99	1.35	1.08
ASS	argininosuccinate synthase	7.61	**8.66**	1.89	1.84	1.04
AVD	avidin	1008.15	753.66	15.15	10.10	0.74
C1QA	complement C1q subunit α	2.54	2.29	3.23	2.45	1.52
C1QB	complement C1q subunit β	2.56	2.34	1.28	2.25	1.53
C1QG	complement C1q subunit γ	3.63	3.00	3.00	3.04	2.44
CCDC86	coiled-coil domain containing 86	3.99	**3.04**	2.43	1.85	1.03
CTSB	cathepsin B	2.26	1.96	0.92	1.35	1.01
CTSC	cathepsin C	1.99	1.88	1.75	1.49	0.84
CTSD	cathepsin C	2.11	2.23	1.31	1.65	0.96
CTSS	cathepsin S	2.38	1.61	1.49	1.97	1.10
ERLEC1	endoplasmic reticulum lectin 1	2.65	**4.84**	0.76	1.04	0.94
EXFABP	extracellular fatty acid binding protein	32.46	35.03	50.63	13.15	1.17
FN1	fibronectin	5.60	3.62	0.60	1.12	1.01
FTH1	ferritin heavy chain	4.39	**6.26**	0.70	1.19	1.03
GSTA	glutathione S-transferase α class	5.54	5.16	2.08	1.62	1.41
HMOX1	heme oxygenase 1	2.45	1.86	1.23	1.23	0.91
IGA	immunoglobulin A heavy chain	8.90	**17.58**	3.50	0.84	0.82
IGLC	immunoglobulin λ light chain	12.13	**20.99**	7.09	1.04	0.68
IGM	immunoglobulin M heavy chain	11.11	**19.39**	3.91	1.22	0.65
IGY	immunoglobulin Y heavy chain	9.48	**16.60**	8.51	1.69	0.53
IRG1	immune-responsive gene 1	96.62	**38.94**	83.17	7.26	0.80
LMNB1	lamin B1	2.11	1.67	2.23	1.03	0.87
MD1	Md1, lymphocyte antigen 86	4.76	3.45	0.52	1.04	1.48
MGST1	microsomal glutathione S-transferase 1	6.61	**3.22**	0.89	0.67	1.00
NAV3	NAv3	1.38	**2.01**	0.77	1.00	1.04
OTFB	ovotransferrin BB type	2.36	**3.64**	2.82	1.92	1.86
PRDX4	peroxiredoxin 4	3.80	**9.50**	0.64	1.03	0.99
PRDX1	peroxiredoxin 1	1.98	**3.01**	2.21	1.00	1.18
SAA	serum amyloid A	55.72	51.21	84.63	14.99	1.16
SEC11C	microsomal signal peptidase sec11c	1.72	2.66	0.92	0.91	1.03
SLC35B1	solute carrier family 35 member B1	1.69	**2.39**	0.70	1.00	1.01
TRAM1	translocating chain-associated membr. protein 1	2.02	3.21	0.92	1.13	1.12
TRAP6	trappin-6	22.65	20.21	36.46	38.14	2.09
TXNDC5	thioredoxin domain-containing protein 5	6.35	**11.59**	0.87	1.02	0.96
VHA16	vacuolar H+ ATB synthase 16 kDa proteolipid	3.39	2.70	0.871	1.05	0.99
VIM	vimentin	2.03	**2.40**	0.86	1.17	1.16

Inf – upregulation in the naive infected chickens, Vacc – upregulation in the vaccinated and infected chickens. Upregulation in gene expression in the spleen was determined 4 days after intravenous infection of 42-day-old chickens, upregulation in the cecum was determined 3 or 4 days after oral infection of 1- or 42-day-old chickens, respectively. Background in red color, significant upregulation when compared with the expression in appropriate non-infected controls. Data in bold, significant difference between the expression in the spleen of infected and vaccinated and infected chickens.

### Expression in sorted splenic leukocytes

Some of the genes upregulated after the infection in the above mentioned experiments were previously reported to be expressed by different leukocyte subpopulations [8]–[10]. Furthermore, we have shown that splenomegaly is associated with macrophage and heterophil infiltration after intravenous infection which may influence the total transcription in the spleen [4]. In the next experiment we therefore used cDNA from our previous study [4] to test which leukocyte subpopulations, if any, were responsible for the expression of genes associated with the chicken's response to *S*. Enteritidis.

Five different groups of genes in relation to their basal expression in particular leukocyte subpopulations prior to infection and to their expression profile after the infection were identified as follows: i) genes similarly expressed in all leukocyte subpopulations, ii) genes preferentially expressed in B-lymphocytes, iii) genes constitutively expressed in macrophages, iv) genes inducible in macrophages and v) genes coding for immunoglobulins (Tab. 2 and for total data set see Tab. S4).

**Table 2:** Basal expression of individual genes in sorted leukocyte subpopulations in the absence of infection.

transcript	CD4	CD8	γδ T-cell	B-cell	MΦ
chemokine ah221	0.78±0.64	0.05±0.03	0.33±0.06	1.27±0.48	2.52±1.83
angiogenin / ribonuclease A	0.73±0.37	0.35±0.24	0.37±0.09	14.2±7.6	13.7±5.4
annexin A2	0.47±0.18	0.17±0.03	0.19±0.05	0.89±0.19	18.8±6.1
acid ceramidase	0.41±0.04	0.42±0.02	0.45±0.01	1.11±0.15	2.72±0.34
argininosuccinate synthase	0.35±0.26	0.10±0.06	0.13±0.01	0.47±0.21	14.4±7.0
Avidin	0.03±0.02	0.02±0.02	0.01±0.00	0.02±0.02	4.90±1.70
complement C1q subunit α	0.22±0.14	0.03±0.02	0.11±0.03	0.80±0.43	2.22±1.59
complement C1q subunit β	0.12±0.06	0.01±0.01	0.07±0.01	0.38±0.16	1.07±0.31
complement C1q subunit γ	0.05±0.04	0.01±0.01	0.03±0.03	0.14±0.11	0.51±0.24
coiled-coil domain containing 86	0.07±0.06	0.01±0.00	0.03±0.01	0.19±0.07	1.83±0.47
cathepsin B	0.98±0.43	0.51±0.21	0.52±0.09	1.61±0.72	7.83±3.01
cathepsin C	0.94±0.36	1.30±0.50	0.66±0.30	2.28±0.90	4.60±1.70
cathepsin D	0.48±0.38	0.88±0.25	0.89±0.13	2.45±0.59	4.13±0.57
cathepsin S	3.19±0.98	1.66±0.66	1.76±0.52	5.18±1.70	10.8±2.6
endoplasmic reticulum lectin 1	1.04±0.47	0.92±0.36	0.41±0.14	2.31±0.94	1.21±0.70
extracellular fatty acid binding protein	0.59±0.29	0.61±0.41	0.33±0.12	2.69±1.40	118±43
fibronectin	0.05±0.02	0.03±0.01	0.01±0.01	0.03±0.01	7.44±4.82
ferritin heavy chain	23.3±9.6	23.8±10.8	24.5±3.1	70.9±28.4	179±70
glutathione S-transferase α class	0.11±0.06	0.03±0.02	0.09±0.04	0.23±0.15	5.50±2.30
heme oxygenase 1	0.32±0.12	0.07±0.00	0.21±0.05	0.84±0.02	2.69±0.50
immunoglobulin A heavy chain	0.10±0.08	0.11±0.08	0.06±0.04	0.64±0.34	0.02±0.01
immunoglobulin λ light chain	11.8±3.57	22.0±10.5	8.57±8.01	154±76	1.29±0.64
immunoglobulin M heavy chain	1.69±1.21	2.95±2.45	1.17±0.85	14.8±11.0	0.16±0.12
immunoglobulin Y heavy chain	3.48±1.85	4.23±2.22	1.70±79	4.31±2.67	0.34±0.10
immune-responsive gene 1	0.00±0.00	0.01±0.00	0.00±0.00	0.01±0.00	0.82±0.60
lamin B1	0.13±0.02	0.09±0.01	0.07±0.02	0.14±0.04	0.24±0.03
lymphocyte antigen 86, Md1	0.03±0.01	0.11±0.06	0.04±0.00	0.29±0.16	12.8±4.0
microsomal glutathione S-transferase 1	0.11±0.07	0.17±0.07	0.22±0.07	0.19±0.09	5.38±2.60
NAv3	0.39±0.06	0.29±0.07	0.33±0.10	0.58±0.18	0.64±0.12
ovotransferrin BB type	0.10±0.03	0.05±0.00	0.05±0.02	1.89±1.06	3.34±0.61
peroxiredoxin 4	0.71±0.30	1.01±0.46	0.55±0.18	3.60±1.51	0.92±0.50
peroxiredoxin 1	1.30±0.10	3.10±0.26	1.62±0.14	3.36±0.87	1.91±0.26
serum amyloid A	0.10±0.05	0.07±0.02	0.08±0.02	0.34±0.25	4.07±1.81
microsomal signal peptidase sec11c	0.89±0.40	0.95±0.40	0.65±0.09	1.97±0.62	1.05±0.54
solute carrier family 35 member B1	1.12±0.69	0.94±0.42	0.58±0.06	1.11±0.46	0.72±0.23
translocating chain-associated membr. protein 1	1.27±0.75	1.24±0.53	0.77±0.09	1.53±0.66	1.04±0.58
trappin-6	0.03±0.02	0.05±0.03	0.03±0.02	0.42±0.22	0.48±0.21
thioredoxin domain-containing protein 5	0.30±0.12	0.47±0.25	0.24±0.03	2.36±1.28	0.37±0.23
vacuolar hH+ ATP synthase 16 kDa proteolipid	2.09±0.91	2.20±0.96	1.74±0.12	1.53±1.21	9.17±4.98
vimentin	2.79±0.16	1.13±0.33	0.73±0.22	2.44±0.75	15.7±2.6

Red background – the basal expressions which differed from the expression in the remaining sorted subpopulations, i.e. gene expressions specific for macrophages or B-lymphocytes.

Genes that were equally expressed in macrophages, B-lymphocytes, CD4, CD8 and γδ T-lymphocytes comprised of LMNB1, PRDX1, SLC35B1, TRAM1, and a transcript of an unknown function (NAv3). The expression of SLC35B1 and TRAM1 slightly decreased after *S*. Enteritidis infection in all subpopulations, independent of the previous vaccination status but without reaching statistical significance. Expression of the remaining genes hardly changed in sorted leukocyte subpopulations after the infection.

Prior to the infection, B-lymphocytes were the major producers of PRDX4, SEC11C, ERLEC1 and TXNDC5 and all immunoglobulins. The expression of PRDX4, SEC11C, ERlEC1 and TXNDC5 decreased in expression in B-lymphocytes after *S*. Enteritidis infection suggesting a suppression of common B-lymphocyte function and specialization of B-lymphocyte towards immunoglobulin expression after the infection. Immunoglobulins are described separately due to their unexpected expression profiles (see below).

Genes transcribed constitutively in macrophages were the most numerous and included AH221, ANG, ANXA2, ASAH, ASS, C1QA, C1QB, C1QG, CCDC86, CTSB, CTSC, CTSD, CTSS, EXFABP, FN1, FTH1, GSTA, HMOX1, IRG1, MD1, MGST1, OTFB and VIM. Out of these genes, the basal expression of EXFABP was as high as the expression of macrophage inducible genes after their induction. Upregulation of these genes in the spleen without their induction in their main producers indicates that upregulation in the spleen was caused mainly by infiltration of macrophages with their characteristic expression profile.

**Figure 2:**
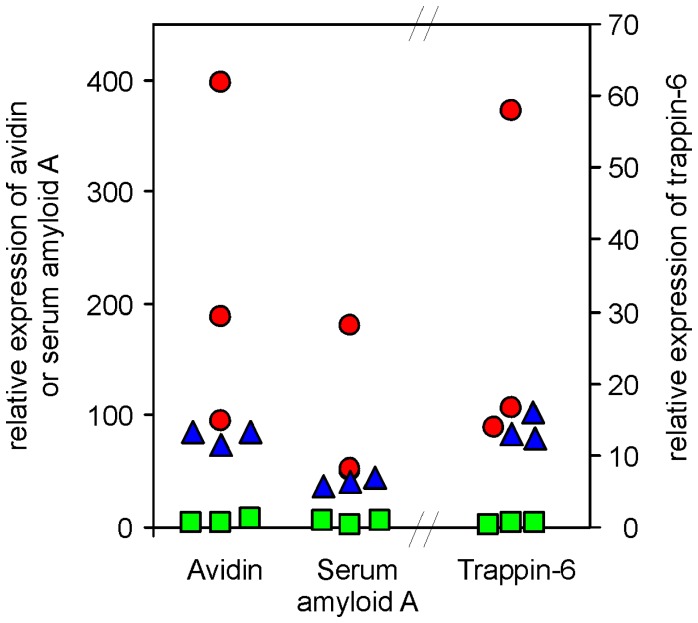
Expression and induction of avidin, serum amyloid A and trappin-6 in macrophages sorted from spleens of non-infected, infected, and vaccinated and infected chickens. Squares –3 non-infected chickens; circles –3 infected chickens; triangles –3 vaccinated and infected chickens.

Macrophage-inducible genes included genes coding for avidin, serum amyloid A and trappin-6 (AVD, SAA, TRAP6). Average upregulations of these genes in macrophages were 46×, 23× and 61×, respectively. We consider these genes as macrophage-inducible despite the fact that none of the comparisons have come out as significant. However, the statistical non-significance was caused mainly by a great variation in the induction rate in macrophages after the infection resulting in a high standard deviation and non-significance when only 3 samples were compared (Fig. 2).

**Figure 3:**
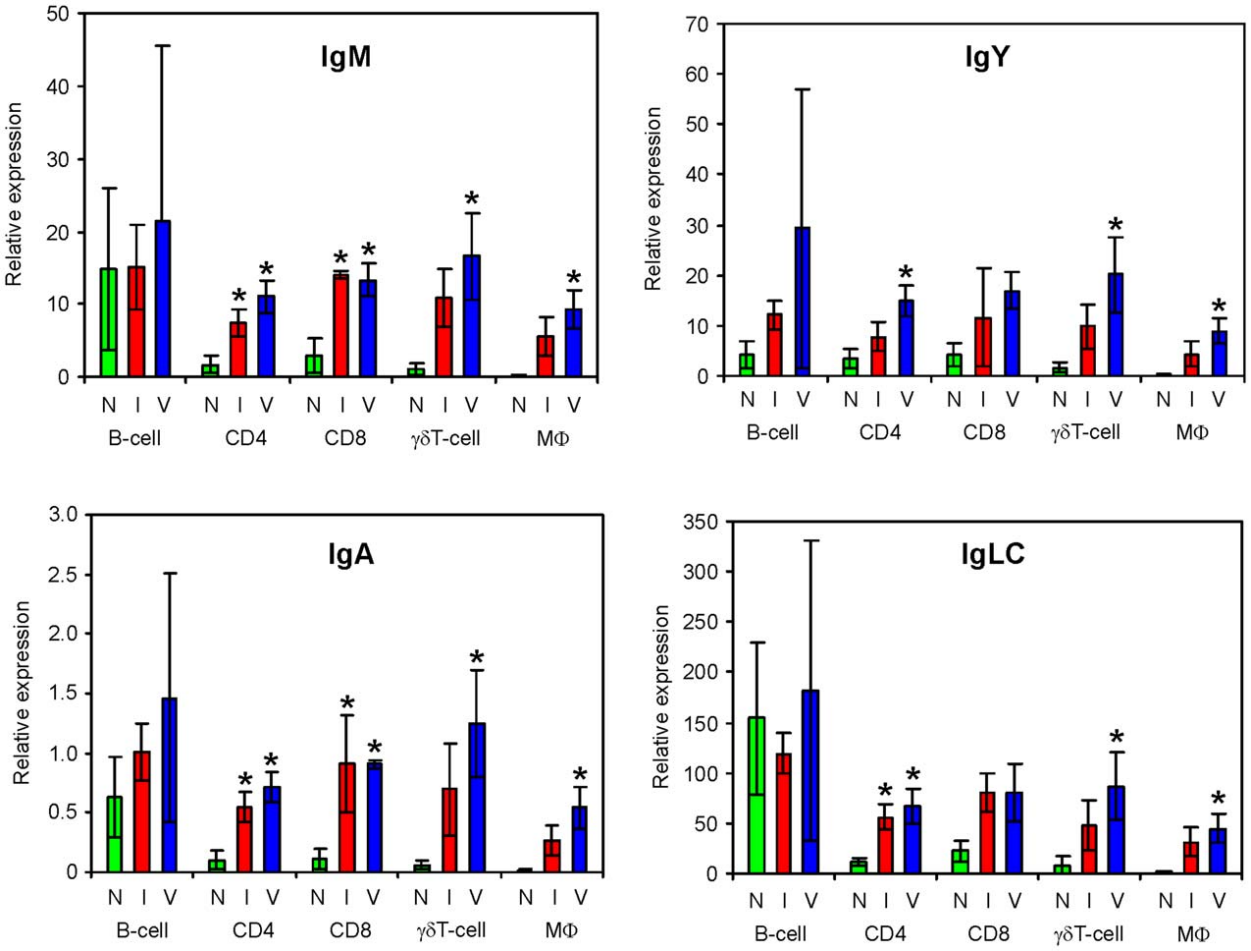
Real-time PCR quantification of transcripts coding for constant parts of immunoglobulins in leukocyte subpopulations. Leukocyte subpopulations were sorted from the spleen of non-infected (N), infected (I) and vaccinated and infected (V) chickens. The infected chickens were i.v. inoculated on day 42 of life and sacrificed 4 days later. IgLC – λ light immunoglobulin chain. Asterisks indicate a significant difference between the expression in sorted leukocyte subpopulations of infected or vaccinated and infected chickens from the same subpopulation of the non-infected chickens (ANOVA, P<0.05).

The last group was formed by the transcripts coding for the constant part of immunoglobulins. Prior to infection, these genes were transcribed the most in B-lymphocytes. However, the transcription of all 4 immunoglobulin genes did not increase after the infection in B-lymphocytes and, instead, there was a significant increase in the abundance of mRNA coding for the constant part of immunoglobulins in both T-lymphocytes and macrophages in response to the infection (Fig. 3). Consequently, the transcription of the constant parts of immunoglobulins in T-lymphocytes and macrophages after the infection reached nearly the same level as in B-lymphocytes.

### Expression of newly identified genes in the cecum

Since intravenous infection can be considered as a rather artificial route of infection, in the next step we verified our results using cDNAs from the cecum of chickens orally infected on the day of hatching and sacrificed 3 days later [3]. Out of the 40 genes identified as upregulated in the spleen, 15 genes were significantly induced also in the cecum (Tab. 1). These included all 4 immunoglobulin genes, and TRAP6, AVD, SAA, AH221, EXFABP, IRG1, C1QA, ANG, LMNB1, OTFB and PRDX1.

One of our aims was to find markers for the protection of vaccinated chickens. In the last verification experiment we therefore used cecal cDNA from 46-day-old chickens, vaccinated and infected, together with appropriate controls [5]. The highest upregulations in naive and orally infected newly hatched chickens were observed for TRAP6, EXFABP, SAA, IRG1, AVD, AH221, C1QG, ANG, C1QA and C1QB. The response of 46-day-old chickens, except for the expression of immunoglobulins, was similar to the response of 4-day-old chickens (Tab. 1). In 42-day-old chickens we could also test the response of chickens which were protected against challenge by previous oral vaccination. The vaccinated and orally challenged chickens responded to the infection only by a significant increase in the transcription of C1QG and MD1, though upregulations of these 2 genes were lower than 2 fold. Unlike the non-vaccinated chickens, genes such as TRAP6, EXFABP, SAA, IRG1, AVD, AH221 or ANG were not significantly induced in the vaccinated chickens and can be therefore used as new markers of vaccination status, in addition to culture detection of the challenge strain or cytokine (e.g. IL-8, IL-17 or IFNγ) targeted real-time PCR [1], [5], [11].

**Figure 4:**
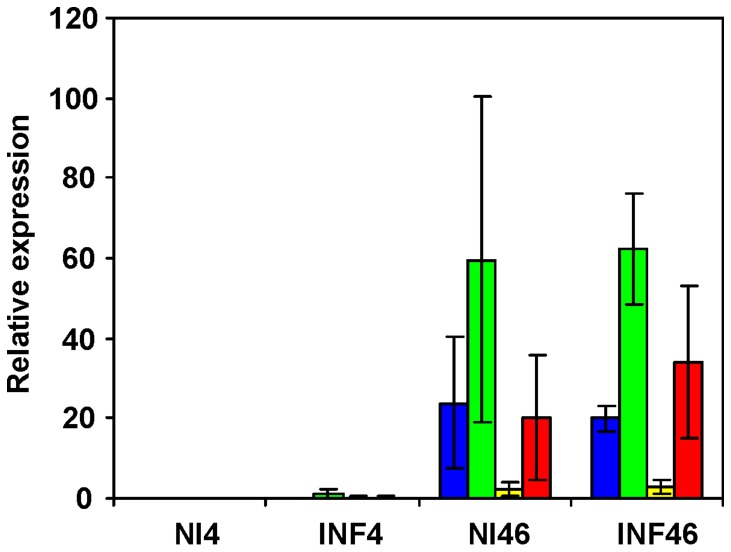
Expression of genes encoding constant parts of immunoglobulins in the cecum of 4- and 46-day-old chickens. NI4 – expression in the cecum of 4-day-old, non-infected chickens, INF4 – expression in the cecum of 4-day-old chickens orally infected with *S*. Enteritidis on day 1 of life, NI46– expression in the cecum of 46-day-old, non-infected chickens, IFN46– expression in the cecum of 46-day-old chickens orally infected with *S*. Enteritidis on day 42 of life. Blue columns – IgA transcript, green – Ig λ light chain transcript, yellow – IgM transcript, red – IgY transcript.

Genes encoding immunoglobulins were not induced in the cecum of 42-day-old chickens after the infection with *S*. Enteritidis. However, when the expression levels in 4- and 46-day-old chickens were compared, the basal expression of immunoglobulins in the cecum in the older chickens, though no longer inducible, reached a considerably higher expression level than in the 4-day-old chickens (Fig. 4). This also means that a gradual increase of immunoglobulin gene transcription must have occurred sometime between day 4 and day 46 and this process was accelerated by *S*. Enteritidis infection in the young chickens.

### Avidin in the chicken response to S. Enteritidis infection

Since avidin was the most inducible gene in the spleen after *S*. Enteritidis infection, in the last experiments we tested its potential role in the defense against *S*. Enteritidis infection. First we tested the direct antibacterial effect of avidin on *S*. Enteritidis but avidin did not affect *S*. Enteritidis growth in LB broth up to 2.5 mg/ml concentration (data not shown).

**Figure 5:**
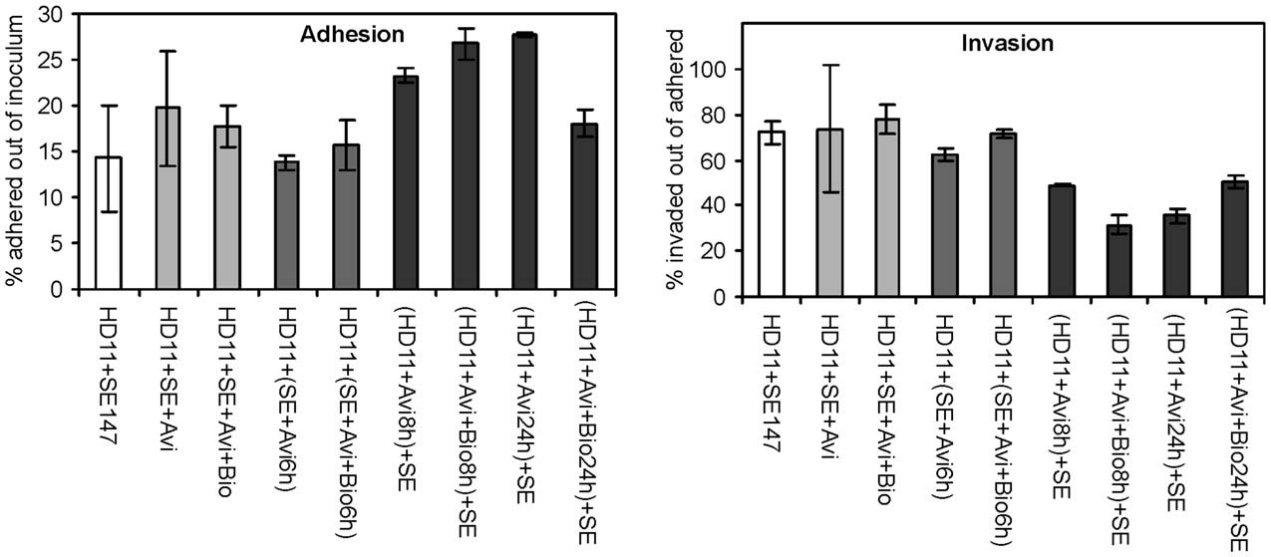
Influence of avidin and biotin on the ability of *S*. Enteritidis to adhere to and invade the HD11 chicken macrophage-like cell line. Parentheses indicate whether the pretreatment with avidin and/or biotin was done on the HD11 cell line or *S*. Enteritidis. Grey scaling is used to simplify individual group differentiation. When the group of all avidin treated HD11 cells was compared with all the remaining experiments in which the cell culture was not pretreated, the comparison came out as significantly different by a t-test with p<0.05, both in adhesion and invasion assays. Avi – avidin, Bio – biotin, SE – *S*. Enteritidis.

Next we tested whether avidin may influence phagocytosis and/or invasion of *S*. Enteritidis into the HD11 macrophage-like cell line. When the HD11 cells were pretreated with avidin prior to *S*. Enteritidis infection, a higher adhesion but lower invasion of *S*. Enteritidis was recorded when compared with the adhesion and invasion into avidin non-treated cells (Fig. 5). On the other hand, pretreatment of *S*. Enteritidis prior to the adhesion and invasion assay did not result in any difference from the assay performed in the absence of avidin (Fig. 5).

Finally we tested whether avidin may protect chickens against *S*. Enteritidis challenge *in vivo*. The chickens were intravenously administered avidin to reach 3 µg of avidin per gram of body weight and, 8 hours later, half of the chickens were intravenously challenged with *S*. Enteritidis. Four days later when the chickens were sacrificed, we did not record any differences in total bacterial load in the liver or spleen (not shown). Similarly, we did not record any differences in the composition of splenic leukocytes (B-lymphocytes, CD4, CD8 and γδ T-lymphocytes, and macrophages) after avidin treatment determined by flow cytometry (data not shown). The only significant differences as a result of avidin administration were the differences in IgM and PRDX1 expressions in the spleen of avidin treated and *S*. Enteritidis infected chickens compared with the chickens infected by *S*. Enteritidis without avidin treatment. The differences were numerically quite low; a 2 fold increase in the transcription for IgM and around 1.5 fold increase in PRDX1 in avidin pretreated chickens. We therefore concluded that avidin likely has functions different from a direct effect on the immune response and chicken resistance to *S*. Enteritidis.

## Discussion

First of all we have to stress that designations and functions of the majority of the genes identified as responding to *S*. Enteritidis infection in this study are based mostly on their sequence similarities to different GenBank entries, rather than their proven function in chickens. The majority of these genes have never been associated with *S*. Enteritidis and chickens although some of these transcripts were described as responsive to *Salmonella* in other experimental animals [12] or were characterized as LPS inducible or as belonging among acute phase proteins. This is true mainly for genes coding for serum amyloid A, avidin, immune responsive gene 1 or extracellular fatty acid binding protein [8], [13]–[17]. The main motif of the immune response to the i.v. infection with *S*. Enteritidis was LPS inactivation, which was also supported by MD1 induction leading to a decrease in TLR4-LPS responsiveness in HEK293 cells [18] or increased proliferation of B-lymphocytes [19], [20]. In agreement with this observation, three classes of heavy immunoglobulin chains (IgM, IgG and IgA) together with a λ light chain were induced after *S*. Enteritidis infection. The rapid increase in immunoglobulin mRNA in both the vaccinated and non-vaccinated chickens 4 days post infection indicates that the antibody response might be a T-cell-independent response to the LPS [21] consistent with our previous reports on the rapid increase of anti-LPS antibodies in the chicken serum after intravenous infection [5]. Furthermore, the spleen also responded by an increased transcription of all 3 subunits of the C1q complement complex which binds to the conserved domains of IgG and IgM in a complex with antigen. Several types of cathepsin proteases were induced, as well as TRAP6, a protease inhibitor, likely protecting host tissues against activity of its own proteases released during inflammation [22], [23]. Six genes could be characterized as having a detoxification function (glutathione S-transferase α class, microsomal glutathione S-transferase 1, peroxiredoxin 1, peroxiredoxin 4, thioredoxin domain-containing protein 5, endoplasmic reticulum lectin 1), most of them dismutating reactive oxygen species. Oxidative burst by phagocytes is decreased also by SAA binding of LPS [15]. Finally, the restoration of damaged tissues during the initial immune response by angiogenesis [24], [25], for example, was induced as early as 4 days post infection as documented by an increased expression of angiogenin, annexin a2, fibronectin and ferritin heavy chain.

Most of the genes identified in this study were associated with macrophages or B-lymphocytes whilst T-lymphocytes were not involved in the response to i.v. infection with *S*. Enteritidis to such an extent that would affect transcription on a level of the whole spleen. Interestingly, not all genes inducible on the level of total spleen were inducible also in the sorted leukocytes. There are two, mutually non-exclusive explanations for this observation. First, there might be other cells in the spleen, which express the genes not inducible in the sorted leukocytes tested in this study. Our unpublished data show that heterophils are responsible for a high expression of serum amyloid A, trappin-6 or extracellular fatty acid binding protein. Secondly, the observed upregulation in the spleen but not in the sorted leukocytes can be explained by an extensive infiltration of particular cell types such as macrophages with their own constitutive gene expression profile. Their numerical increase, despite the absence of induction, then leads to changes in the net gene transcription of the whole spleen. In the case of avidin, its expression was induced in sorted macrophages approx. 46×, and macrophages increased from 0.5% to 10%, i.e. 20× after the infection as determined by flow cytometry (see ref. [4] and data not shown). The combination of the induction and infiltration, i.e. 46×20 = 920, is in agreement with the total increase in avidin expression in the spleen determined by real-time PCR to be 1008× (Tab. 1).

Quite unexpected profiles were observed for the expression of immunoglobulins. Whereas these genes were constitutively expressed in B-lymphocytes, they were inducible in all T-lymphocyte subpopulations and even macrophages. We excluded that this could be caused by contamination of sorted T-lymphocytes and macrophages by B-lymphocytes since in such a case, the contamination with B-lymphocytes should influence the expression of immunoglobulins also in T-lymphocytes and macrophages from the non-infected chickens. We also excluded the possibility that surface markers used for macrophage and T-lymphocyte sorting might be present on the surface of clonally expanding B-lymphocytes as there would have to be B-lymphocytes positive for CD4, CD8, TcR1 and macrophage surface markers. Finally, we also consider as unlikely the hypothesis that the real-time PCR detected target sequences are similar to but different from the immunoglobulin transcripts since the same results were obtained for 4 independent targets and the transcription of the light chain was equivalent to the sum of the expression of transcripts for heavy chains, as one would expect. Finally, comparing the expression in different tissue samples, IgY and IgA dominated over IgM in the cecum whilst IgY and IgM dominated over IgA in the spleen, which confirmed the correctness of the real-time PCR results. This means the increase in the expression of immunoglobulins in T-lymphocytes and macrophages is likely correct whereas its biological function remains unknown. However, several older papers described that T-lymphocytes and macrophages were capable of transcription of immunoglobulin genes though the function might be different from immunoglobulin secretion [26]–[29].

Since intravenous infection is quite an artificial mode of infection, we also tested the expression of the newly identified genes in the cecum of orally infected chickens. AVD, SAA, TRAP6, AH221, EXFABP and IRG1 were also highly upregulated in the cecum after oral infection, both in 4- and 46-day-old chickens. Although some of these proteins, e.g. avidin and serum amyloid A, have been known for a long time, their biological function is poorly understood. AH221, also known as CCLi10 or predicted C-C motif chemokine 3, is produced by macrophages as a chemotactic protein during inflammation. The function of chicken IRG1 (immune responsive gene 1) is even less clear (predicted chicken IRG1 was 74% identical and 83% similar to murine immune responsive gene 1 at amino acid level). IRG1 is induced by LPS or *Mycobacterium tuberculosis* in murine bone marrow derived macrophages independent of TLR2 or TLR4 sensing of pathogen-associated molecular patterns [30] but the biological relevance of this is unknown. On the other hand, although trappin-6 has never been studied in chickens and its identification in this study was based only on sequence similarities (42% identical and 58% similar to bovine trappin-6 at amino acid level) [31], its likely function is the protection of the host's extracellular proteins from degradation by its own proteases such as neutrophil elastase or proteinase 3 [22], [32]. We have shown that trappin was expressed by macrophages and our unpublished data show that it is also highly transcribed in heterophils. This can serve as additional, though indirect, evidence that the trappin 6-like transcript codes for a functional protease inhibitor. Chicken macrophages and heterophils therefore likely release extracellular proteases [23] and in parallel also protease inhibitors protecting their own tissues from proteolytic degradation.

Chicken EXFABP was first characterized as a protein capable of binding unsaturated fatty acids with an unknown role in chondrocyte development [9], [33], [34]. EXFABP also stimulates cell proliferation and its suppression results in apoptosis [17]. Interestingly, recent reports showed that quail lipocalin Q83 and chicken EXFABP, which share 88% similarity, have dual binding capacities and besides the fatty acid binding capability, they can also bind bacterial siderophores [35]–[37]. Consequently, chicken EXFABP inhibited the growth of *E. coli* in iron-limited media *in vitro* [37] and this likely affects the multiplication of gram negative bacteria after LPS sensing also *in vivo*. Interestingly, since *Salmonella* produces glycosylated enterochelin resistant to the activity of Lcn2 in mice [12] and likely also EXFABP in chickens, it may obtain a growth advantage over the rest of the cecal microbiota.

The contribution of serum amyloid A and avidin, although known for a long time, to the defense against *S*. Enteritidis is unclear. Serum amyloid has been shown to bind LPS and cholesterol [14], [15]. Avidin, besides biotin binding, was shown to block chondrocyte proliferation without any effect on their differentiation [13]. This means that these two proteins together with EXFABP may decrease LPS concentration and the associated inflammatory response, suppress cell proliferation during the inflammatory response and provide host cells with fatty acids, cholesterol and biotin [14]. This might be consistent with our results showing a decrease in the phagocytosis of avidin treated HD-11 cells *in vitro* and the absence of any direct antimicrobial activities of chickens administered with avidin prior to *S*. Enteritidis infection.

Taken together, 6 transcripts (AVD, SAA, TRAP6, AH221, EXFABP and IRG1) therefore as central to the control of the chicken response to *S*. Enteritidis infection, both in the spleen during the systemic presence of *S*. Enteritidis and in the cecum during a cecum-localized infection. Moreover, as the transcription of these genes increased in the cecum of naive chickens after *S*. Enteritidis infection but essentially did not change in the vaccinated chickens, these genes may be used as new markers for chicken response to *Salmonella* infection.

## Materials and Methods

### Ethics Statement

The handling of animals in the study was performed in accordance with current Czech legislation (Animal protection and welfare Act No. 246/1992 Coll. of the Government of the Czech Republic). The specific experiments were approved by the Ethics Committee of the Veterinary Research Institute (permit number 48/2010) followed by the Committee for Animal Welfare of the Ministry of Agriculture of the Czech Republic (permit number MZe 1226).

### Experimental animals, sample collection and pyrosequencing

Three samples of spleen from ISA Brown chickens were used for RNA isolation. The first spleen originated from a 46-day-old, non-infected chicken, the second one from a non-vaccinated chicken infected intravenously with *S*. Enteritidis on day 42 of life, and the third one from a chicken which was orally vaccinated on day 1 and day 21 of life with *S*. Enteritidis 147 ΔSPI1 mutant, and intravenously challenged on day 42 of life with wild type *S*. Enteritidis [5]. Both infected chickens were sacrificed 4 days after the challenge. Approximately 30 mg of spleen was collected from each chicken and stored in RNALater (Qiagen) at −70°C for RNA isolation.

Total RNA was isolated with RNeasy Mini Kit (Qiagen) followed by mRNA Isolation Kit (Roche) to enrich the total RNA for mRNA species. cDNA libraries from the three spleen samples were prepared with the GS Rapid Library Preparation Kit (Roche) and approx. 2 molecules of cDNA per bead were used in emulsion PCR. All steps of the cDNA library preparation for sequencing were performed with the GS Junior Titanium series kits according to the manufacturer's instructions (Roche). The pyrosequencing was performed with the GS Junior 454 sequencer (Roche) and each of the 3 samples was sequenced in a separate sequencing run.

### Data analysis

In the first step we used the De Novo Assembler software provided with the GS Junior. This software was used to assemble the chicken spleen transcriptome using the sff files generated as an output of sequencing of each of the 3 samples. Out of this analysis we took the 454Isotig.fna file which contained sequences of all transcripts assembled and identified after merging the data from all 3 splenic samples. The data present in this file were used in two independent analyses. Firstly, the 454Isotig.fna file was uploaded into Blast2GO software to associate each transcript with a gene designation and gene ontology classification according to the GeneBank [38]. In a second independent analysis we used the 454Isotig.fna file as a reference file using GS Reference Mapper software provided with GS Junior and analyzed the sff sequencing files of each of the 3 samples. Data from the ReadStatus.txt output file after this analysis allowed us to determine the number of reads in each of the 3 sequenced samples matching different splenic transcripts present in the 454Isotig.fna reference file. Only reads longer than 100 nt and matching the reference transcripts by more than 60% of their sequence were included for the quantification of gene expression. These values were arbitrarily selected but effectively excluded short repetitive sequences or sequences with a polyA motif. The last negative selection was applied using chicken rRNA gene sequences as an exclusion filter. Transcripts predicted as being downregulated after *S*. Enteritidis infection included those for which we identified at least 10 independent reads in the transcriptome of non-infected spleen and when the fold downregulation was 3-fold or higher.

A slightly less stringent selection criteria were applied for the transcripts predicted as being upregulated after *S*. Enteritidis infection since these were subjected to verification by real-time PCR. The tentatively upregulated transcripts were chosen as those which were present either in the infected or vaccinated and infected spleen at 10 or more reads and the calculated induction was at least twofold. In addition, for further analysis we also included the transcripts which were detected once or not at all in the transcriptome of the non-infected spleen but were recorded in the transcriptome of either the infected or vaccinated and infected spleen at least 8 times.

### Real-time PCR verification of the pyrosequencing data

Real-time PCR was used for the verification of the pyrosequencing data. Primers for the quantification of expression real-time PCR were designed using Primer3 software [39] (for primer sequences see Tab. S5). First we used the same cDNAs as in the pyrosequencing reactions followed by an additional 2 spleen samples for each group of chickens available from our previous study [4]. After such screening we reduced the number of tested genes to those in which the real-time PCR confirmed the results from pyrosequencing which meant at least a twofold induction and statistical significance or threefold average upregulation without reaching statistical significance – the latter case, however, happened only once. Using the reduced set of real-time PCRs, we finally determined the gene expression in i) sorted splenic leukocyte subpopulations using already available cDNA [4], ii) the cecum of 4-day-old orally infected chickens using cDNA from our recent paper [3], and iii) the cecum of 46-day-old orally infected chickens using cDNA from another recent paper of ours [5].

Real-time PCR was performed in 3 µl volumes in 384-well microplates using QuantiTect SYBR Green PCR Master Mix (Qiagen) and a Nanodrop II Stage pipetting station (Innovadyne) for PCR mix dispensing. The amplification of PCR products and signal detection were performed using a LightCycler II (Roche) with an initial denaturation at 95°C for 15 min followed by 40 cycles of 95°C for 20 s, 60°C for 30 s and 72°C for 30 s. Each sample was subjected to real-time PCR in duplicate and the mean Ct value of the duplicates was used for subsequent analysis. The Ct values of the genes of interest were normalized (ΔCt) to an average Ct value of three house-keeping genes (GAPDH, TBP and UB) and the relative expression of each gene of interest was calculated as 2^−ΔCt^. These expression levels were used for data analysis and are presented in the tables and figures as average ± SD. As an alternative, the fold upregulation calculated as a ratio of averages of infected to non-infected samples are shown. However, also in this case, the significance of these upregulations was calculated by comparing the expression levels, i.e. the 2^−ΔCt^ values determined for the individual samples.

### Biological testing of the transcriptome data

Since avidin was identified as the gene with the highest induction after *S*. Enteritidis infection, we tested whether it has a direct role in the chicken defense against *S*. Enteritidis. First we tested whether avidin might be of direct antimicrobial effect to *S*. Enteritidis. Twofold serial dilutions of avidin in LB broth were prepared in 96-well microplates starting with a 2.5 mg/ml avidin concentration. A fresh 18-hour-old culture of *S*. Enteritidis was inoculated into the wells of the microplates and the growth of *S*. Enteritidis was visually inspected after a 24 hour incubation at 37°C.

The influence of avidin and its ligand biotin on the invasiveness of *S*. Enteritidis into the chicken macrophage-like cell line HD11 was tested using standard gentamicin protection. The HD11 cell line was pre-treated for 8 and 24 hours with avidin (20 µg/ml) or avidin and biotin (biotin was used at 50 ng/ml), prior to the addition of *S*. Enteritidis at multiplicity of infection of 10. In parallel, *S*. Enteritidis was exposed to avidin or avidin and biotin for 6 hours prior to to the addition to HD11 cell line. The last experimental group consisted of *S*. Enteritidis added to the HD11 cells at the same time as avidin or mixture of both. Controls included the adhesion and invasion assay performed in the absence of avidin or biotin. Adhesion was determined 1 hour after addition of *S*. Enteritidis to the HD11 cell line and the invasion after an additional hour of incubation in the presence of 100 μg/ml gentamicin. In all the cases, the numbers of intracellular (or adherent) bacteria were determined after lysis of the cell line with 1% Triton X-100 and plating tenfold serial dilutions on LB agar plates.

In the last experiment we tested the protective effect of avidin *in vivo*. Four groups of 8-week-old chickens were included in this experiment, each consisting of 6 chickens. The first group was used as a non-treated control. Chickens in group 2 were given 0.1 ml of 20 mg/ml avidin intravenously into the wing vein to reach an avidin concentration of 3 µg per gram of body weight [40]. These chickens were sacrificed 8 hours later. Chickens in group 3 were intravenously administered avidin into the left wing vein like the chickens in group 2 and 8 hours later, they were intravenously infected into right wing vein with 10^7^ CFU of *S*. Enteritidis in 0.1 ml PBS. Chickens in group 4 were given only *S*. Enteritidis like the chickens in group 3. All infected chickens in groups 3 and 4 were sacrificed 4 days later. During the post mortem analysis, *S*. Enteritidis counts in the spleen were determined in all infected chickens. Spleen samples were placed into RNALater and stored at −70°C. RNA purification, reverse transcription into cDNA and real-time PCR were performed as described previously [3]–[5]. Finally, in 3 chickens of all 4 groups, the cellular composition of splenic leukocytes was characterized by flow cytometry exactly as described earlier [4].

### Reproducibility and statistics

454 pyrosequencing was considered as an initial screening and was performed on individual samples from the spleen of a non-infected chicken, an infected chicken and a vaccinated and infected chicken. An initial verification of the 454 screening by real-time PCR was done with 3 chicken samples in each group. However, when performing the experiments with avidin administration, an additional 6 spleen samples after i.v. challenge and 9 from the non-infected chickens were analyzed with the same results.

Data for expression in the cecum were obtained from 9 infected and 10 non-infected 4-day-old chickens and from 6 non-infected, 6 infected and 6 vaccinated and infected 46-day-old chickens.

The adhesion and invasion assays were performed in duplicate and the experiment was performed on two independent occasions with similar results.

The last experiment with avidin administration was repeated on two independent occasions with 3 chickens in each group, except for the non-infected chickens for which we included two different control groups in the repeated experiment, the first one not being treated at all and the second one treated intravenously with sterile water used for dissolving the avidin. This means that the data were calculated for 6 chickens in each group treated with avidin or *S*. Enteritidis and 9 controls without any avidin or *S*. Enteritidis inoculation.

Statistical significance was calculated using GraphPad Prism software (GraphPad Software, Inc.), either by ANOVA followed by a post-hoc Tuckey's test or a t-test as indicated in the text. Differences with p<0.05 were considered as significant.

## Supporting Information

Table S1 **List of all transcripts identified by 454 pyrosequencing.**
(XLS)

Table S2 **List of gene predicted as downregulated in the spleen after i.v.** ***S*** **. Enteritidis infection based on 454 pyrosequencing.**
(XLS)

Table S3 **List of gene predicted as upregulated in the spleen after i.v.** ***S*** **. Enteritidis infection based on 454 pyrosequencing.**
(XLS)

Table S4 **Expression of genes upregulated in the spleen after i.v.** ***S*** **. Enteritidis in sorted splenic leukocytes determined by real time PCR.**
(XLS)

Table S5 **List of primers used in this study.**
(XLS)
